# Towards Intelligent Drug Design System: Application of Artificial Dipeptide Receptor Library in QSAR-Oriented Studies

**DOI:** 10.3390/molecules23081964

**Published:** 2018-08-06

**Authors:** Andrzej Bak, Violetta Kozik, Malgorzata Walczak, Justyna Fraczyk, Zbigniew Kaminski, Beata Kolesinska, Adam Smolinski, Josef Jampilek

**Affiliations:** 1 Department of Synthesis Chemistry, Faculty of Mathematics, Physics and Chemistry, University of Silesia, Szkolna 9, 40007 Katowice, Poland; violetta.kozik@us.edu.pl; 2 Institute of Organic Chemistry, Lodz University of Technology, Zeromskiego 116, 90924 Lodz, Poland; malgorzata.walczak@p.lodz.pl (M.W.); justyna.fraczyk@p.lodz.pl (J.F.); zbigniew.kaminski@p.lodz.pl (Z.K.); beata.kolesinska@p.lodz.pl (B.K.); 3 Central Mining Institute, Gwarkow 1, 40166 Katowice, Poland; asmolinski@gig.katowice.pl; 4 Department of Pharmaceutical Chemistry, Faculty of Pharmacy, Comenius University, Odbojarov 10, 83232 Bratislava, Slovakia

**Keywords:** artificial dipeptide library, PX2 inhibitors, IVE-PLS, CoMSA, stochastic model validation

## Abstract

The pharmacophore properties of a new series of potential purinoreceptor (P2X) inhibitors determined using a coupled neural network and the partial least squares method with iterative variable elimination (IVE-PLS) are presented in a ligand-based comparative study of the molecular surface by comparative molecular surface analysis (CoMSA). Moreover, we focused on the interpretation of noticeable variations in the potential selectiveness of interactions of individual inhibitor-receptors due to their physicochemical properties; therefore, the library of artificial dipeptide receptors (ADP) was designed and examined. The resulting library response to individual inhibitors was arranged in the array, preprocessed and transformed by the principal component analysis (PCA) and PLS procedures. A dominant absolute contribution to PC1 of the Glu attached to heptanoic gating acid and Phe bonded to the linker *m*-phenylenediamine/triazine scaffold was revealed by the PCA. The IVE-PLS procedure indicated the receptor systems with predominant Pro bonded to the linker and Glu, Gln, Cys and Val directly attached to the gating acid. The proposed comprehensive ligand-based and simplified structure-based methodology allows the in-depth study of the performance of peptide receptors against the tested set of compounds.

## 1. Introduction

Drug discovery is a complex process that is focused on the optimization of the molecular interactions between a drug molecule and a biomolecular target. The contacted drugs or, more generally, ligands (in Latin ‘ligare’ means ‘tie’) and macromolecules initiate the signals that control substantial effects of living organisms [1]. Finding balance between expected hit →lead→seed→drug candidate potency and its ADMET-related properties to identify high-quality marketed drug molecules seems like searching ‘a needle in a haystack’ that lurks behind the complex property space that is delimitated by the complex nature of ligand-target interactions, which are ruled by inter-/intra-molecular phenomena [2,3]. In order to discard compounds that have undesirable biological/physicochemical features according to the ‘fail-early fail-cheaply’ paradigm and to reach more successful ‘stories’ in rational drug design, the quantitative conversion of the structural/feature-based descriptors into an ADMET-tailored molecular potency is highly desirable as a ‘heuristic guide’ that relates the in vitro or in vivo parameters to calculable physicochemical properties [4]. A key question that confronts computational chemists is how to quantitatively transform a myriad of feature-based descriptors a priori calculated for the compounds into ADMET-related molecular potency. From among optimistic approaches, modelling multidimensional quantitative structure-activity relationship (mD–QSAR) techniques seem to be pragmatic provided that a congeneric series of compounds is processed [5]. Conceptually, a classical QSAR strategy is based implicitly or explicitly on the principle of similarity, where ‘congeneric’ series of molecules show similar pharmacological profile [6]. On the other hand, a variety of observed guest-host interactions is a potential pitfall while modulating desired ADMET-related properties. Simple Hantzsch-type descriptors are not often sufficient to provide the ‘physically realistic’ descriptive and/or predictive rules how to ‘navigate’ through factual/virtual chemical space (CS = FCS∪VCS). Hence, the ‘indirect’ ligand-based and ‘direct’ protein-based multidimensional in silico protocols produce informative descriptors to capture more interpretable SAR trends potentially useful in the optimization phase of the novel drug candidates.

In receptor-independent (RI) procedures, the ‘reverse image’ of the target binding geometry is provided in the form of pharmacophore maps displaying the spatial arrangements of crucial guest-host interaction patterns. Typically, ligand-based 3D methods employ the comparison of molecular shape, since molecules can be characterized as having ‘skin’ of a particular thickness. In particular, 3D-QSAR shape-based methodologies with a classical comparative molecular field analysis (CoMFA) and comparative molecular surface analysis (CoMSA) diffused quickly into computational/medicinal chemistry getting more and more popular in the drug design area [7]. In receptor-dependent (RD) approaches, specific requirements for efficient guest-host interactions are deduced directly from the protein-binding cavity in target-guided QSAR procedures [8]. Considering long-term aspiration, these ligand-based procedures should be combined with structure-based ones or homology models in order to retrieve a detailed insight into mutual drug-target interactions.

In absence of an empirically specified receptor structure, a valid estimation of the host binding geometry should be supplied using flexible sensors or molecular probes [9]. It seems that a set of artificial receptors (AR) can compose a diagnostic platform capable to selectively interact with small chemical reagents under controlled conditions [10]. In consequence, a natural question appears about preferable combinations of building blocks that comprise a ligand and/or a target structure. Is it possible to tailor theoretically and/or empirically a protein cavity to enhance the target druggability in fragment-based domain design? Generally, comprehensive answers to the above questions still exceed our capabilities despite many efforts directed towards this issue. In response to this pressure, the potential of artificial receptors that are smaller and therefore structurally less complicated by definition as a model system has been reported recently [11]. In fact, the ensemble of artificial biosensors formed by *N*-lipidated peptides immobilized on cellulose was arranged in the form of a probe matrix to recognize the ligand shape, size and polarity [12]. Due to high peptide and lipid fragment flexibility, the shape, size and polarity of the binding cavity can be adequately adjusted reflecting the induced fit phenomenon observed in real biological systems; however, the extremely simplified fragment-based representation of an artificial cleft emphasizes the more local nature of the intricate drug-receptor recognition pattern in physiological fluids. The allocation of various structural and physicochemical attributes corresponding to hydrogen bond donors/acceptors and lipophilic motifs supplemented with π-donors/acceptors enabled to form a linear structure, namely a matrix of podands in such a way that a flexible *N*-lipopeptide fragment is preceded by relatively rigid aromatic rings. In other words, the binding cleft comprises tethered fragments of walls that are generated from aromatic rings expanded with a flexible peptide fragment and closed with the lipophilic chain of lipidic zipper creating a molecular trap to effectively match a guest molecule. The regular pattern of immobilized podands on the surface of the solid carrier not only generates a multitude of feature library components by themselves, but also provides data about the relationships between them [13]. A comprehensive target-guided guest response can work as a diagnostic fingerprint useful in monitoring changes of the structure-affinity pattern.

In fact, the most challenging are attempts to rationalize interactions with an unknown molecular target. For classic bi-functional alkylating nitrogen mustards, anticancer activity is associated with the process of crosslinking of DNA strands [14,15]. Our studies on mono-, di-, and tri-functional mustards attached to the triazine ring revealed that the first ones are the most active among them [16,17]. Obviously, mono-functional alkylating agents cannot be engaged in the cross-linking process. A strongly diversified mode of action proposed for the anticancer agent constructed on the 1,3,5-triazine scaffold prompted us to perform preliminary SAR studies using the library of randomized receptors that were prepared using all proteinogenic amino acid residues [18]. After the analysis of interactions between ligands and receptors that strongly bind the most active of them, a set of the preferred amino acid residues was specified. The identified residues can be useful while screening active sites of already known targets of anticancer triazine derivatives. Moreover, experimental findings can provide clues to the composition of the molecular target for mono-alkylating (2-chloroethyl) triazine derivatives. In the case of 2,4,6-triamino-1,3,5-triazine derivatives with various amino substituents, the proposed mechanism of action represents an interaction with the active cleft of ATPase, DNA topoisomerase IIα or p21-activated kinase 4 [19,20,21]. Considering that the suggested activity of 2,4,6-triamine-1,3,5-triazine derivatives is associated in most cases with enzymes/receptors crucial for building blocks of nucleic acids or energy carriers also containing a purine moiety, it is suspected that the 2,4,6-triamine-1,3,5-triazine core may mimic this heterocyclic ring. One of such receptors could be a P2X receptor—a membrane receptor present in cells of the variety of tissues. P2X receptors represent new therapeutic targets forming non-selective cation channels with a unique trimeric architecture gated by extracellular ATP [22,23,24]. In response to ATP binding, the receptor conformation is switched from ‘closed’ to ‘opened’ allowing the permeation of ions (especially Ca^2+^) through the membrane that leads to the depolarization of cells. In fact, P2X receptors mediate a variety of physiological processes (e.g., neuro/immunomodulation, inflammation, synaptic transmission); therefore, the development of novel therapeutic agents targeting the binding pocket is valid from the point of view of anticancer activity.

There were two principal objectives of the current investigation. First, it is of interest to perform a ligand-based investigation with a comparative study of molecular surface using comparative molecular surface analysis (CoMSA) to produce a pharmacophore pattern for a new set of 20 derivatives as potential anticancer agents. On the other hand, it should be strongly emphasized that we focused more on the development of ‘descriptive’ pharmacophore models within the congeneric series of compounds than ‘predictive’ rules with strictly specified domain of applicability; however, the similarity of compounds was scrutinized using the principal component analysis (PCA) approach for the pool of generated descriptors. The probabilistic pharmacophore geometry using the stochastic model validation (SMV) approach indicated the relevant contributing factors for the anticancer agent activity profile. Secondly, we focused on the interpretation of noticeable variations in the potential selectiveness of individual inhibitor-receptor interactions; therefore, the combinatorial library of artificial dipeptide receptors (ADR) was designed, synthesized, empirically examined and arranged in the form of a probe matrix in the pseudo structure-based examination of ligand responses.

## 2. Results and Discussion

### 2.1. Library of Docked Anticancer Active Triazine Derivatives

The collection of docked anticancer active triazine derivatives was split into various (sub)groups taking into account the chemotype of the compounds (alkyl, aromatic, cyclic), their lipophilic character (logP ≤ 5, logP > 5) and activity profile (active, non-active) as shown in Table 1. The general pattern of the anticancer compounds comprises the *N*-(2-chloroethyl)piperazine moiety and, in the overwhelming amount, a methoxy substituent on the triazine ring [13,16,17]. The third substituent of the triazine ring was amine, amino acid and peptide derivatives. With the exception of compounds **15** and **20**, all tested molecules contained two amino substituents mimicking the structure of 2,4,6-amino-1,3,5-triazine derivatives with various amino substituents. Compounds were divided into the following chemotypes: derivatives containing aromatic amine residues (**1**–**3**), aliphatic amines (**4**–**6**), amino acid residues (**7**–**9**, **17**, **18**), dipeptide derivatives (**10**, **12**, **13**, **19**) and derivatives containing two moieties *N*-(2-chloroethyl)piperazine and aromatic amine residues (**14**, **15**, **20**) or the methoxy substituent (**11**, **16**). The investigated compounds can also be subdivided in relation to the hydrophobicity and hydrophilicity. Derivatives containing amino acid residues/dipeptides showed increased polarity in comparison to derivatives containing aromatic or aliphatic (cyclic and acyclic) substituents.

### 2.2. In Silico Evaluation of In Vitro Anticancer Activity Using CoMSA and SMV Approach

The general purpose of the ligand-based examination was the comparative shape analysis via molecular surface variations (electrostatic/steric features) using the CoMSA approach to model in vitro activity observed for the new set of 20 derivatives as potential anticancer agents. Hence, we investigated the pharmacophore properties of the target series using a coupled neural network and the partial least squares method (PLS) with iterative variable elimination (IVE). The outcomes of inhibiting potency modelling (IC_50_) were correlated with surface descriptors considering multiple training and test subsets or (in)dependent variables used. Unfortunately, the performance of the inhibitory profile for the entire set of molecules in the training dataset is not satisfactory for CoMSA models ($q_{\mathrm{cv}}^{2}$ < 0.5), irrespective of the map size (20 × 20–50 × 50). A question appears regarding the relevance of simulated and real model predictability that cannot be only tested by simple assessment of data fitting. Consequently, we performed external validation with splitting the molecule subset into a set of training/test collections to evaluate the model predictive ability with SDEP and $q_{\mathrm{test}}^{2}$ statistics. The CoMSA performance for models divided arbitrarily into training/test subsets in 2:1 ratio (14/6) ranked according to the molecule inhibitory activity (IC_50_) was scrutinized as well. In all cases, the best $q_{\mathrm{cv}}^{2}$/$q_{\mathrm{test}}^{2}$ outcomes indicated fairly poor model abilities accompanied with low model predictive power. Moreover, we employed the Kennard-Stone algorithm on the dependent variable to divide representatively the ensemble of data into training/test subsets; however, the statistical characteristics of CoMSA models were not noticeably improved. The obtained findings confirmed that the division of objects into training/test subgroups having a restricted set of molecules, for which activity data are available *a priori*, is not a trivial issue, and a model with predictive capability should by all means characterize SAR relations inherent in the test set. Accordingly, an additional approach, namely SMV, has been used as a kind of perturbation procedure to examine the structure of data. Hence, we investigated the variations of the statistical estimators during CoMSA inhibitory activity modelling, as the initial population of 20 molecules was recurrently sampled into 14/6 training/test subseries (fraction 2/3 to 1/3). Usually, the inspection of all possible combinations is resource/time demanding; however, in this case, it was technically viable to inspect the whole pool of systematically generated training/test populations ($C_{20}^{6}$ ≈ 4 × 10^4^). The observed profile for $q_{\mathrm{cv}}^{2}$ vs. $q_{\mathrm{test}}^{2}$ distribution confirms the intuitive interpretation of the $q_{\mathrm{cv}}^{2}$/$q_{\mathrm{test}}^{2}$ fluctuation pattern where the regions of higher modelling ability within the training set can be depicted for the pIC_50_ profile ($q_{\mathrm{cv}}^{2}$ ≥ 0.75). Obviously, the preferential selection of molecules into the training set, which easily fit into the model, can be accompanied with a decrease in the predictive power for the remaining ones, which confirms the dichotomic nature of $q_{\mathrm{cv}}^{2}$/$q_{\mathrm{test}}^{2}$ parameters, where a high value of $q_{\mathrm{cv}}^{2}$ does not automatically imply high model predictability [25]. In consequence, we should beware of q^2^, because the great advantage of the QSAR/QSPR paradigm does not lie in extrapolation [26]. The compound selection into the training/test subsets was investigated, while sampling the best CoMSA models ($q_{\mathrm{cv}}^{2}$ ≥ 0.75), indicating the test molecules (**9**, **10**, **13**–**15**, **17**) that disrupt the modelling efficiency. Consequently, the comparative molecular field analysis (CoMFA) and CoMSA performance for models divided according to the specified training/test selection improved considerably ($q_{\mathrm{cv}}^{2}$ = 0.66 vs. $q_{\mathrm{cv}}^{2}$ = 0.72).

**Figure 1:**
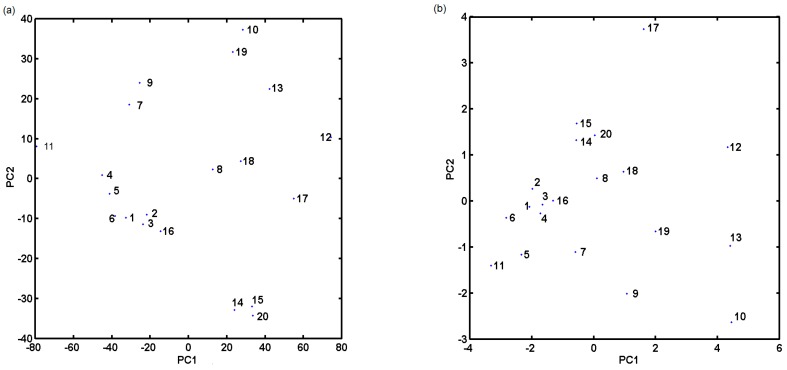
Projection of molecules described by Dragon (**a**) and by Sybyl (**b**) parameters on plane defined by PC1 and PC2.

**Figure 2:**
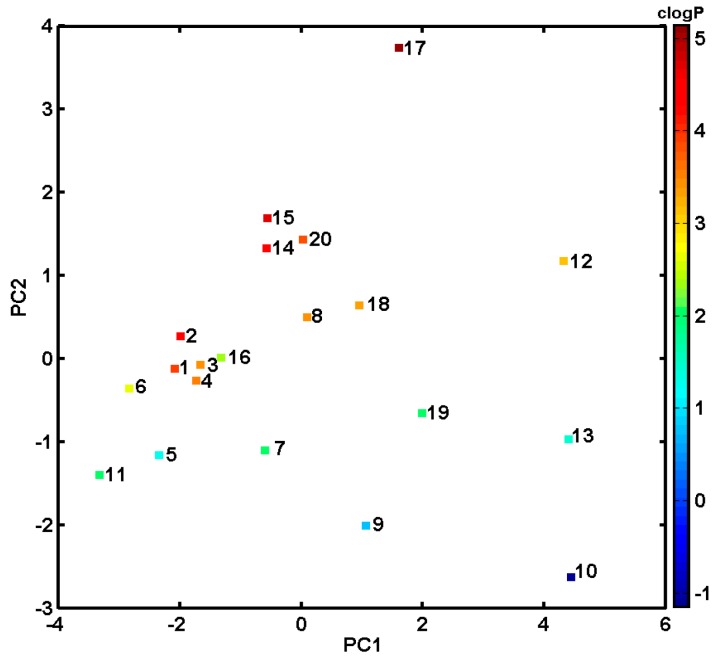
Projection of molecules described by Sybyl parameters on plane defined by PC1 and PC2 with their color-coded lipophilicity profile.

Drug-likeness is an idea that drug compounds differentiate from other molecules in their physical properties; therefore, a priori calculation of molecular descriptors (topological descriptors, structural fingerprint keys and other properties) is crucial for compound’s bioavailability and, hence, critical for the prospective drug candidate. Hence, a PCA technique for an ensemble of descriptors derived using Dragon 6.0 package was employed to the analysed compounds. All columns with constant or nearly constant values (standard deviation < 10^−4^) and with missing values have been eradicated from the initial number of selected parameters (4885) at the pre-processing stage resulting in the final set of 2797 descriptors. The final dataset was arranged in matrix X_20×2797_ with rows representing molecules (called objects) and columns representing numerical parameters (called variables) for further analysis. The efficient investigation of (dis)similarities between the studied objects requires simultaneous consideration of all calculated parameters. Thus, PCA was employed to visualize relevant differences in the performance of investigated molecules with respect to their structure and inhibitory profile. The analysis was performed for centred and standardized data, since the studied data library includes parameters of various orders of magnitude. The percentage of modelled variance was applied in the determination of the number of significant components (PCs). The PCA model with first three PCs described 79.78% of the total data variance, while the first two PCs accounted for 72.79%. Projecting objects on the planes defined by a few first principal components allows an interesting observation of the data structure. The analysis of score plot PC1 vs. PC2 in Figure 1a indicates that, basically, anticancer derivatives can be classified into groups according to the first principal component (describes 57.90% of the total variance). Roughly speaking, PC1 reveals variations between objects (molecules), where the test compounds are generally grouped together (PC1 > 20), confirming the trends observed in CoMFA/CoMSA modelling. Noticeable variations between molecules **11** and **12** encoded by a set of descriptors (parameters) confirm their mutual structural dissimilarity. Similarly, nine parameters (variables) calculated by the Sybyl software were gathered including count, volume, surface, Lipinski’s rule of five (Ro5) and lipophilicity descriptors (Table S1 in Supplementary Materials) to investigate variations within the ensemble of 20 anticancer derivatives. The compression of data slowly increased with the number of PCs that were included. The first two PCs account for 86.28% of the total data variance and it increases to ca. 93.28% for the third PC. The projection of objects on the plane defined by PC1 vs. PC2 component (Figure 1b) confirmed the tendency observed previously to cluster compounds of the test subpopulation (PC1 > 0). Interestingly, distant object no. **17** can be regarded as an outlier to the remaining ones along PC2 since it violates Ro5. Moreover, the inspection of compound lipophilicity colour-coded accordingly to calculated values of clogP detected that objects are clustered along PC2 (PC2 < −1) more or less according to their lipophilicity profile as illustrated in Figure 2. The projection of variables on the plane defined by first and second loadings for Dragon and Sybyl parameters is presented in Figure S1 (Supplementary Materials).

**Figure 3:**
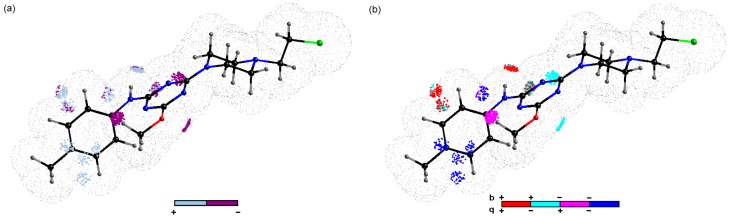
Molecular plots show spatial sectors of the greatest contribution to inhibitory potency retrieved from the best CoMSA model. Colours code the sign of influence (**a**). Four possible combinations of mean charge and correlation coefficient are colour-coded for charge descriptors (**b**). Compound **2** was plotted as a reference molecule.

We applied the subsequent level of variable elimination in order to generate meaningful and predictive CoMSA models. The recursive IVE-PLS algorithm was employed as a filter to specify structural descriptors having the highest individual weightings to the biological activity [27]. Generally, a slight improvement of the $q_{\mathrm{cv}}^{2}$ performance was observed when the column from the data matrix that is assigned with the lowest value of *abs*(*mean*(*b*)/*std*(*b*)) was extracted. The moment of $q_{\mathrm{cv}}^{2}$ deterioration indicates the number of relevant columns; therefore, the backward column elimination is recurrently repeated, until the optimal number of variables included in the model is accomplished. A simplified visual inspection of the generated pharmacophore pattern gives a clear 3D picture of the regions that should be modified to modulate the biological activity [28]. The sign of influence is colour-coded, indicating not only regions with positive and/or negative activity contribution, but also four possible combinations of the mean charge/correlation coefficient. The bright polyhedra in Figure 3a specifies 3D areas where an atom or a substituent is foreseen to be positioned in order to increase the activity of the compound, while dark spheres indicate patterns detrimental for the inhibitory potency, mainly due to steric hindrance or electrostatic factors. In particular, some regions with suggested unfavourable contribution appear in the close proximity to the negatively charged oxygen atom and hydrogens of the metoxy group directly attached to the triazine motif. It seems that an increase of the negative charge on oxygen by donor substituents can be detrimental for the anticancer activities of the examined compounds as indicated by the corresponding regression coefficient pool in Figure 3b. Moreover, the obtained findings demonstrate the importance of the area occupied by the positively charged hydrogen atom bonded to the nitrogen atom as a significant factor interacting non-covalently with the macromolecular site (hydrogen bond donor). The displayed arrangement of the bright polyhedra (Figure 3a) illustrates the significance of negatively charged atoms (mainly oxygen atoms) of the side chain as shown in Figure 3b. From the structural point of view, electronic parameters can notably effect the process of IC_50_ modulation, mainly due to inductive or resonance effects, as depicted by the activity of molecules **8**, **9**, **12**, **13** and **18**.

It should be emphasised that the SMV protocol for the pharmacophore visualization based on the CoMSA modelling with satisfactory statistical parameters provides a spatial distribution of chemical groups/atoms potentially important for increasing/decreasing the activity profile of prospective anticancer agents.

### 2.3. Probing Artificial Dipeptide Receptor Library

**Figure 4:**
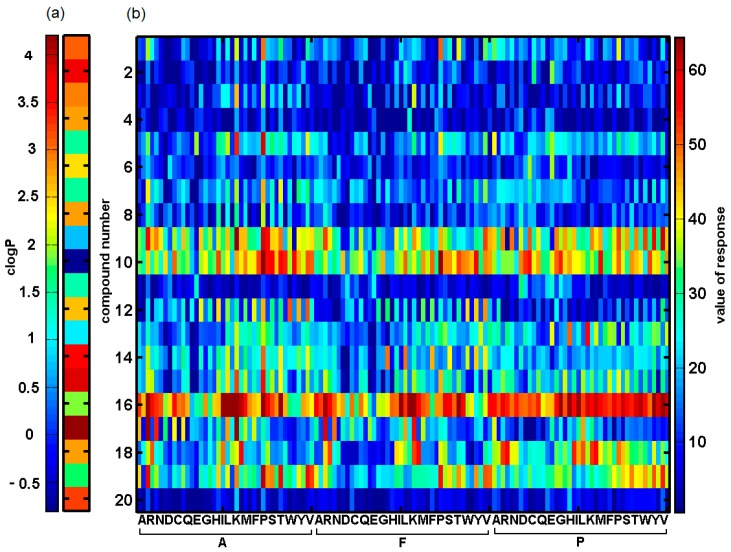
Calculated lipophilicity profile (**a**) and ligand response values (**b**) obtained for set of 120 receptors (columns) and 20 compounds (rows).

The general objective of this pseudo structure-based examination was the comparative study of ligand responses from the artificial receptor library in order to probe the prospective purinoreceptor (P2X) inhibitors. In fact, we focused on the interpretation of noticeable variations in the potential selectiveness of individual inhibitor-receptor interactions due to their physicochemical properties. The experimental data contains a set of responses from the artificial library for 20 probe compounds that exhibit different structural and physicochemical properties. The library is an ensemble of artificial receptors, each of which is a strain of two amino acids (X–Y) and lipid residues composed of heptanoic and decanoic acids (denoted as H and D, respectively). The entire set of 20 amino acids was taken into account in the library design for residues directly attached to carboxylic acids (X = A, R, N, D, C, Q, E, G, H, I, L, K, M, F, P, S, T, W, Y, V), while one out of three (Y = A, F, P) was examined in the position of binding to the linker, thus resulting in the total of 120 (2 × 20 × 3) artificial receptors. Amino acid blocks and gating acids (clogP_H_ = 2.41 vs. clogP_D_ = 3.93) were selected in order to provide a fairly robust measure for the diverse ADR constructs that should imitate a real receptor structure. These are attached to cellulose and spatially distributed. The interaction between the examined compounds and the library of artificial receptors is visualized using the competitive adsorption-desorption mechanism of a reporter dye (Brilliant Black) used as an Indicator Displacement Assay, IDA and is monitored using a Sunrise Microplate Reader with the Magellan Software [29]. A more detailed description of the construction of the artificial receptor library is beyond the scope of this article, but can be found elsewhere [9,10,11,12,13]. The data set was then prepared for further analysis; hence, it was organized into a matrix containing 120 artificial receptor responses in columns for a set of 20 tested compounds in rows as illustrated in Figure 4b.

**Figure 5:**
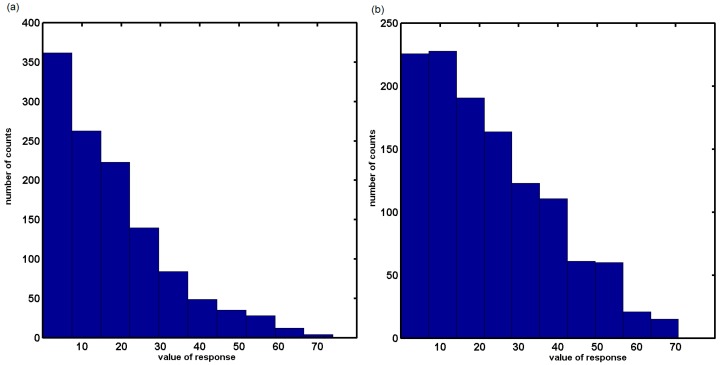
Histogram of response values for set of receptors with heptanoic (**a**) and decanoic (**b**) gating acids.

The examination of the response value indicated that all tested compounds responded with positive values resulting in colour changes. The non-direct method was used to probe the affinity of the compounds vs. the receptor library, i.e., the library was first saturated with ligands and then coloured with a dye preparation. The final colour was recalculated to the affinity of the compounds vs. individual receptors as the difference with the coloration of the receptor with the reporter dye but without a ligand with the maximal value of 73.9 and minimal of 0.001. It seems that compounds annotated with clogP < 2 reveal higher values of the response as shown in Figure 4a. The distribution of the response values that were obtained for all receptors with heptanoic and decanoic gating acids probed for the 20 ligands is presented as histograms in Figure 5.

**Figure 6:**
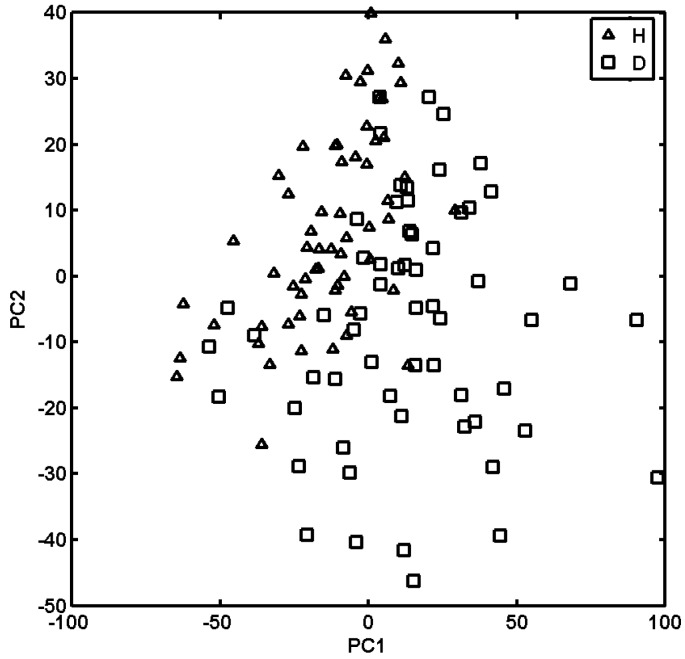
Projection of receptors on plane defined by first and second principal components with type of lipid residue (heptanoic and decanoic).

The elongation of the acid chain produces stronger guest-host interaction (the response values > 50 for 7.3% of decanoic vs. 4.2% of heptanoic gating acids) that can be roughly correlated with the acid lipophilicity profiles. Regarding the multivariate nature of the binding issue, PCA was employed to illustrate crucial variations in the performance of the artificial receptors with respect to their binding strength. The analysis was performed for centred and transposed data X_120×20_. The PCA model with three PCs described 68.53% of the total data variance, while the first two PCs account for 51.98% of the total data variance. First, let us examine the influence of the receptor design with respect to two different lipid residues, heptanoic (H) or decanoic (D), on its binding selectivity as illustrated in Figure 6.

**Figure 7:**
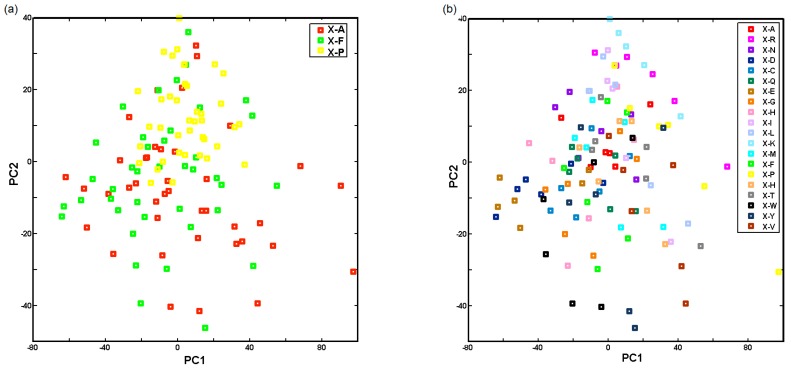
Projection of receptors on plane defined by first and second principal components with type of first (**a**) and second (**b**) residue used to build receptor structure.

The analysis of the score plot that was constructed as projections of the experimental data on the individual lipid acid indicated that, basically, these residues could be classified into two groups regarding their structural/lipophilic features. Accordingly, heptanoic-terminated receptors (clogP_H_ < 2.5) are roughly located in the area of PC1 < 0 and PC2 > 0, while decanoic ones (clogP_D_ > 2.5) are evenly distributed within the rest quadrants. The largest differences in the interaction strengths between a probe compound and a given receptor are observed for the loadings characterized by the largest absolute values. Interestingly, it was noticed that along PC2, some of the largest positive values of loadings were yielded for probe compound **16** annotated with high receptor library responses (see Figure 4b), especially with Lys attached to the heptanoic residue. On the other hand, compound **11** is described by a negative value on loading PC1 contributing mainly to Glu linked to heptanoic acid. Basically, compound **11** produces a pretty poor receptor library response being annotated with poor anticancer activity (IC_50_) as well. Let us now examine interaction strength as a function of the amino acid fixed as the first and the second residues within the receptor constructed. Projections of all receptors onto a plane defined by the first and the second scores are presented in Figure 7, while individual amino acids fixed in positions 1 and 2 are coded by different colours.

Roughly speaking, proline-based receptors are separated from the alanine/phenyloalanine ones along PC2 > 0 (Figure 7a) indicating structural incoherencies between these two groups of residues—sometimes proline is named as imino acid. No clear relationship is observed for amino acids arranged at position 2 that are directly attached to gating lipid residues; however, basic amino acids (Arg, His, Lys) are vaguely separated from the acidic and aromatic ones along PC1 as shown in Figure 7b.

**Figure 8:**
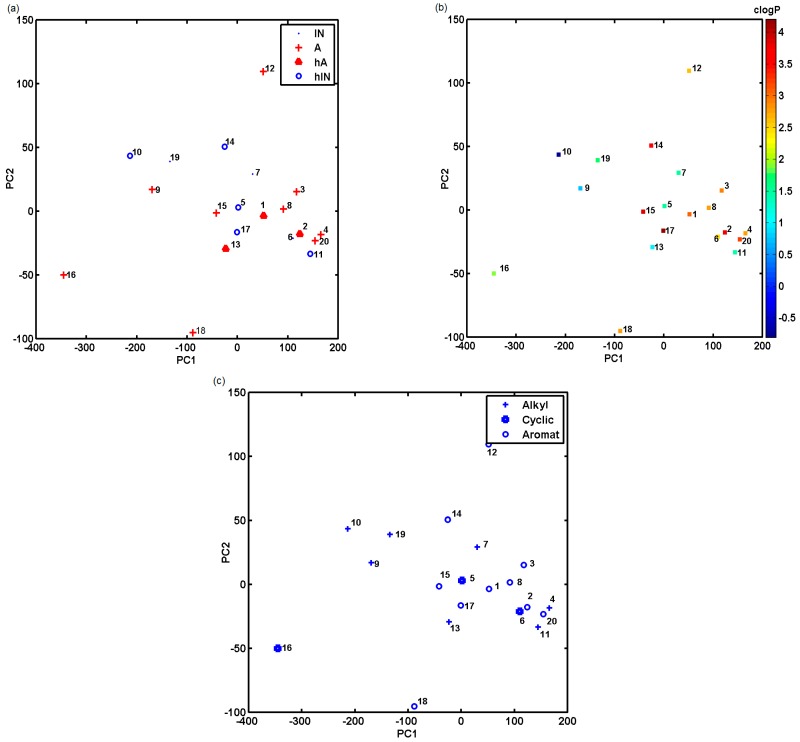
Projection of compound set on plane specified by first and second components with their activity profile (**a**), calculated lipophilicity values (**b**) and chemotype structure indicated (**c**).

Basically, it can be expected that interactions between the target and the host ligands will be associated with the chemical structure of the receptor and the probe compound. Therefore, structurally similar ligands, namely, chemotypes should be retained similarly (comparable interaction strengths) by similar receptors, while diverse ligand structures would have different binding strengths, which would also induce dissimilar receptor answers. We also investigated the data set matrix with a collection of testing compounds as input objects (20 × 120). In this case, the first two principal components explained approximately 75.36% of the total data variance, while the set of three principal components accounted for nearly 84.58%. The analysis of the score plot PC1 vs. PC2 with the indicated activity compound profile (hA: IC_50_ < 25, A: 25 < IC_50_ < 50, IN: 50 < IC_50_ < 100, hIN: IC_50_ > 100) revealed the potency for the separation of biotypes along PC1, where highly active (hA) and active (A) ones are generally grouped together (PC1 > −100) as depicted in Figure 8a.

It is worth noting that compounds annotated with pretty high ADR response (**9**, **10**, **16** and **19**) are characterized by large negative values on the first principal component (PC1 < −100) accompanied with lower values of clogP (Figure 8b). Moreover, the examination of compound chemotypes indicates a similar tendency, especially within the aromatic group specified by higher values of clogP as illustrated in Figure 8c. The investigation of the corresponding loading plot showed a dominant absolute contribution to PC1 of Glu attached to heptanoic gating acid and Phe bonded to the linker *m*-phenylenediamine/triazine scaffold. This is an important finding, since Phe is also a genuine ligand counterpart in the natural P2X receptor [30].

At this point, the question appeared whether it is possible to relate ADR response with the compound activity profile (pIC_50_). We noticed the lack of correlation between mean/median values of the ADR response for the entire set of compounds and their activities. Surprisingly, the similar tendency was observed for the ADR response and compound lipophilicity profiles, even within the biologically preferred range of 3 < clogP < 5. It seems that the gating acid behaves similarly to the cell membrane, where ligands, the lipophilicity of which is low enough, cannot come into contact with the high lipophilic barrier of the gating acid, while those with the exceedingly high lipophilicity are trapped by this barrier and cannot penetrate into the ADR cleft. It seems that the library used is not a good model of the binding clefts responsible for the biological activities measured by experimental IC_50_ values, therefore tri-/tetra-peptide models should be examined in the future as well.

On the other hand, let us eliminate inactive compounds—a pretty clear relation can be observed for molecules with higher affinity and mean values of response with the correlation coefficient r of −0.80 and −0.87 for heptanoic and decanoic subgroups, respectively. The response values for the above-mentioned artificial receptors were then correlated with −log(1/activity). A negative r value is due to colour reaction that was applied—the higher the level of activity, the less intense the colour of a probe. The largest negative, but rather low, coefficient for the heptanoyl-Glu-Ala system with r = −0.52 was revealed. Comparable results of correlation were obtained mainly for heptanoyl-Glu/Trp/Phe-Phe (r ≈ −0.45) receptor components.

Basically, informative variable selection is not an indispensable pre-processing procedure to prune the input assemble of generated descriptors; however, it can indicate the combination of ADR receptors that contribute significantly to the activity of compounds. Hence, PLS models were constructed for the centred data with their complexity estimated by the LOO-CV procedure. Considering the number of objects, the maximum number of PLS components was truncated to 7. Thus, columns annotated with the highest stability for each of the randomly chosen models were identified using the IVE-PLS methodology. In general, the elimination of a column characterized by the lowest value of the regression coefficient from the data matrix improved $q_{\mathrm{cv}}^{2}$ until the optimal number of variables included within the model was reached. The performance of PLS models obtained with model elimination provided the approximate value of $q_{\mathrm{cv}}^{2}$ = 0.91 for H and D subsets. The IVE-PLS method indicated receptor systems with predominant Pro units (fraction of selected/all among selections) bonded to the linker, which appeared to be dominant amino acids irrespective of the receptor/compound subseries—Ala and Phe are selected in comparable quantities. Moreover, decanoic acid selection outperforms slightly heptanoic one, which can be related to the acid lipophilicity profile. When we compared the selected amino acids directly attached to the gating acid, we noticed that Glu, Gln, Cys and Val are frequently indicated as significantly contributing the anticancer activities of the analysed compounds. Interestingly, each subset receptor retained by the IVE-PLS contained receptors that were characterized by the highest negative correlation (activity vs. the mean value of response). In this section, we mainly focused on the interpretation of noticeable variations in the potential selectiveness of individual inhibitor-receptor interactions due to their physicochemical properties.

## 3. Experimental Section

### 3.1. CoMFA Analysis

Comparative molecular field analysis (CoMFA) is a well-known methodology capable of simulating the influence of molecular shape on modelling steric (Lennard-Jones) and electrostatic (Coulomb) fields as important intermolecular interactions involved in non-covalent ligand-receptor binding [31]. Basically, the CoMFA approach is based on the assumption that the comparative analysis of spatial patterns generated within the cubic mesh of points which encompasses aligned molecules by means of suitable probes can explain variations in binding affinities or biological activity profiles for a series of congeneric compounds. In fact, the modelling efficiency of electronic and steric potentials in the molecular environment is strictly affected by the selection of a suitable atomic probe (usually positively charged sp^3^ carbon atom) and superimposition applied following the putative pharmacophore hypothesis. Consequently, the distribution of potential values on mesh points relies on the molecular coordinates that is a decisive factor controlling the value of atomic partial charges. The resulting potential energies at each lattice point can be depicted graphically as three-dimensional coloured contour pattern illustrating the spatial areas, where steric hindrance and/or charged substituents enhance or diminish the binding affinity. A conventional imitation of the molecular boundaries with masking explicit shape information using the regular cubic grid lattice and/or molecular surface can be helpful in explaining pharmacological effects; therefore a number of alternative CoMFA-like protocols have emerged recently as well.

### 3.2. CoMSA Analysis

Comparative molecular surface analysis (CoMSA) is a data processing tool based on two-dimensional topology composed of a single neuron layer typically arranged into a hexagonal or rectangular array of nodes with unsupervised learning procedure [32]. The winning and neighbourhood distances precisely specify mutual relations between neurons in the network. The presented *m*-dimensional input vector $x_{s}=\left(x_{s1},\ldots ,x_{\mathrm{sm}}\right)$ is distributed between neurons according to similarity/correlation weight criteria, where similar inputs are located in the same or proximal nodes. A typical competitive Kohonen (KNN) procedure is based on the comparison of the input vector with the corresponding *m*-element weight vector $w_{i}=\left(w_{i1},\ldots ,w_{im}\right)$ that describes each neuron in order to detect the winning one (out_C_), into which a particular input will be projected. The winning neuron is selected by the optimization of the Euclidean distance between weight (*w*) and vector (*x*) according to the following formula:

(1) $$out_{C}=\min[\sum_{i=1}^{m}{\left(x_{i}-w_{ij}\right)}^{2}]$$

where *j* = 1, …, *n* refers to a particular neuron, *n* refers to the number of neurons, *m* is the number of weights per neuron and s indicates a particular input.

In the winner takes most (WTM) methodology, the weights of the winning neuron and its neighbours are then modified to resemble and subsequently attract similar input vectors. While the next input vector is being presented to the network, the entire procedure is repeated. As a matter of fact, self-organizing neural mapping (SOM) is considered as a nonlinear projection tool, which reduces the dimensionality of the input object, e.g., converts 3D objects to 2D, while preserving the topological relationships between the input and output data. Moreover, the trained network can be employed for projections of the specified molecular property prescribed to the input vector with the creation of the planar colour-coded clustering pattern called a feature map. In consequence, the SOM algorithm was used to generate an electrostatic potential map as a two-dimensional topographic pattern receiving the input signals from points sampled randomly at the molecular surface. In such application, the specification of the closest neighbour and then projection of signals into this particular neuron is based on the comparison of each 3-dimensional input vector consisting of *x*, *y*, and *z* coordinates with a 3-element weight vector describing each neuron. The knowledge concerning the shape of the certain molecular surface (template) encoded in the weights of the trained Kohonen network can be used for processing signals coming from the surface of other molecule(s) (counter-template) providing a series of comparative SOM maps to compare/contrast superimposed molecular geometry. A wide range of SOM applications for the compression, classification and visualization of structural data has been described in chemistry, in particular, for two-dimensional mapping of electrostatic potential on three-dimensional molecular surfaces or partial atomic charges for atomic molecular representation [33]. The fuzzification of the molecular representation that improves the compound’s superimposition seems to be of special interest due to a prospectively better description of binding affinity to a putative receptor structure.

### 3.3. PLS Analysis with Iterative Variable Elimination

The partial least squares (PLS) method generates a relationship between dependent variable *Y* and an ensemble of descriptors (independent variables) **X** expressed briefly in the form of the following formula:

*Y* = **X***b* + *e* (2)

where *b* is a vector of regression coefficients and *e* is a vector of errors.

The obtained data were centred and processed by the PLS analysis with the construction of models, the complexity of which was approximated based on the leave-one-out cross-validation procedure (LOO-CV) [34]. A cross-validated leave-one-out $q_{\mathrm{cv}}^{2}$ value for the assessment of the model performance is measured using the following equation:

(3) $$q_{cv}^{2}=1-\frac{\sum_{i}^{m}{\left(obs_{i}-pred_{i}\right)}^{2}}{\sum_{i}^{m}{\left(obs_{i}-mean\left(obs_{i}\right)\right)}^{2}}$$

where *obs* is an assayed value; *pred* is a predicted value; *mean* is the mean value of *obs*, and *i* refers to the object index, which ranges from 1 to *m*.

The standard deviation of the error of prediction (SDEP) and $q_{\mathrm{test}}^{2}$ parameters were estimated to evaluate the quality of external predictions as follows:

(4) $$\mathrm{SDEP}=\sqrt{\frac{\sum_{i}^{n}{\left({\mathrm{pred}}_{i}-{\mathrm{obs}}_{i}\right)}^{2}}{n}}$$

(5) $$q_{\mathrm{test}}^{2}=1-\frac{\sum_{i}^{n}{\left({\mathrm{obs}}_{i}-{\mathrm{pred}}_{i}\right)}^{2}}{\sum_{i}^{n}{\left({\mathrm{obs}}_{i}-\mathrm{mean}\left({\mathrm{obs}}_{i}\right)\right)}^{2}}$$

where n is the number of objects in the test set.

Redundant variables can deteriorate the outcomes of regressive analysis; therefore, a reduction in the number of uninformative variables might considerably improve PLS models. Despite the fact that variable elimination in PLS modelling is a complex issue, a few algorithms have been introduced recently, e.g., iterative variable elimination (IVE-PLS) which was successfully employed in multidimensional (mD-QSAR) methods [35,36,37]. The iterative IVE-PLS procedure is used in the current calculations as an enhancement of the single-step UVE algorithm for identification of variables to be eliminated [38]. Briefly, the whole algorithm is composed of the following stages:

Stage 1. Standard PLS analysis with LOO-CV to assess the performance of the PLS model;
Stage 2. Elimination of the matrix column with the lowest *abs*(*mean*(*b*)/*std*(*b*)) value;
Stage 3. Standard PLS analysis of the new matrix without the column cancelled in stage 2;
Stage 4. Recurrent repetition of stages 1–3 to maximize the LOO $q_{\mathrm{cv}}^{2}$ parameter.

### 3.4. PCA Analysis

The mapping of molecular diversity forming the *infinite* chemical space (CS) into the corresponding biological or property space generally requires multi-dimensional descriptor representations [39]. A specified molecule might be represented by a set of structural (S) and physicochemical (P) properties organized in a vector which represents an object in the CS. The molecular distribution of empirically (factual chemical space, FCS) and virtually (virtual chemical space, VCS) generated compounds might be graphically investigated using, e.g., a linear projection procedure called principal component analysis (PCA). The restrained set of orthogonal descriptors (principal components, PCs) being a linear combination of weighted input vectors forms a basis for lower-dimensional space, while maintaining the input space topology [40]. The projection of multi-dimensional objects into two/three-dimensional PC space enables the diversity exploration by calculating distance metrics. The PCA model decomposes information contained in the data matrix into principal components—scores and loadings. The score matrix contains information about any similarities among the data objects, while the loading matrix allows similarities among the variables and their role in the construction of the given principal component to be studied. The PCA model with *f* principal components for a data matrix *X* can be presented as follows:

*X* = *TP^T^* + *E* (6)

where *X* is a data matrix with *m* objects and *n* variables, *T* is the score matrix with dimensions (*m* × *f* ), *P^T^* is a transposed matrix of loadings with dimensions (*f × n*) and *E* is a matrix of the residual variance (*m × n*) that is not explained by the first *f* principal components. The PCA usually reduces the number of variables while retaining most of the original information.

### 3.5. Artificial Dipeptide Receptor Library Synthesis

**Scheme 1:**
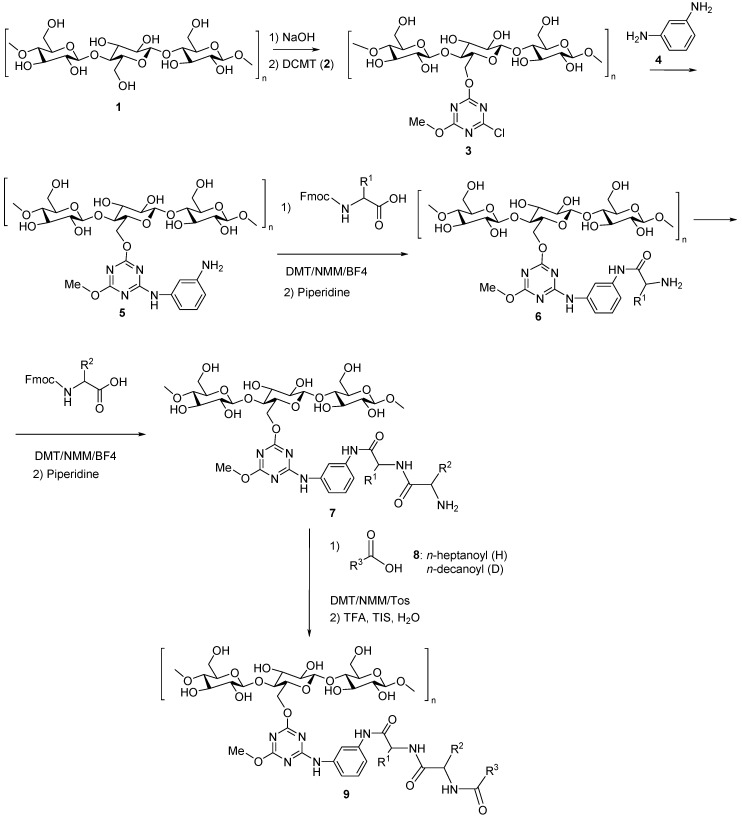
Synthetic procedure used for preparation of ADR using SPOT methodology.

The artificial dipeptide receptor (ADR) arrays were synthesized on cellulose plates according to the SPOT methodology with the modified structure and properties of the linker [41]. Amino acid blocks and gating acids were selected in order to provide a fairly robust measure for diverse ADR constructs that should imitate real active residues of receptors; therefore, a randomized dipeptide library was applied, which allows systematic (all natural amino acids at the *N*-terminal position) study of the effect of the peptide fragment structure on the binding to/interaction with biologically active compounds. The library of molecular receptors was prepared from a π-acceptor, π-donor aromatic rings, peptide and lipid fragments. It was found in previous studies that the presence of all of these was vital for the binding of ligands [42]. Each copy of the array was prepared on a separate page. The library was synthesized using an automated procedure (Scheme 1) starting with the immobilization of 2,4-dichloro-6-methoxy-1,3,5-triazine (**2**) (used as a π-acceptor) on 26 cellulose membranes that were used as solid supports **1**. The incorporation of *m*-phenylenediamine (**4**) (introduced as a π-donor) generated a relatively rigid nucleophilic scaffold, which enabled further surface modification by acylation of compound (**5**) with *N*-protected amino acids. The peptide fragment was synthesized under classic solid phase peptide synthesis conditions using Fmoc protected amino acids (**6**) and 4-(4,6-dimethoxy-1,3,5-triazin-2-yl)-4-methylmorpholinium tetrafluoroborate (DMT/NMM/BF_4_) as a coupling reagent [43]. Six membranes were used in the tests to monitor the progress of the synthesis. Twenty membranes were used for the preparation of the randomly designed array of 120 dipeptides *N*-acylated with heptanoic acid and/or decanoic acid. Side chain functional groups were protected by acid labile protecting groups. After each coupling stage, the Fmoc group was cleaved by the treatment with a 25% solution of *N*-methylpiperidine in DMF. Heptanoic acid (H) and decanoic acid (D) were activated with 4-(4,6-dimethoxy-1,3,5-triazin-2-yl)-4-methylmorpholinium 4-toluenesulphonate (DMT/NMM/Tos) before being used for the *N*-lipidation of the immobilized peptides. Side chain protecting groups were removed using the standard procedure that includes the treatment of functionalized cellulose with TFA in the presence of triisopropylsilane (TIS) [44]. The array of artificial peptide receptors (**9**) was synthesized in this way. The structures of the ADR library (**9**) are presented in Table S2 of Supplementary Materials. All ligand docking experiments were performed using a brand new array of receptors.

The details of synthetic procedure are given below. Immobilization of 2,4-dichloro-6-methoxy-1,3,5-triazine (DCMT): 10 sheets (10 × 10) of Whatman-Chr 1 filter paper were immersed in 1 M NaOH (40 mL) and gently shaken for 15 min. An excess of solution was removed, then the wet paper sheets were soaked in suspension of finely grounded sodium bicarbonate in 1 M solution of 2,4-dichloro-6-methoksy-1,3,5-triazine (DCMT) (6.8 g) in THF (40 mL) and again gently shaken for 60 min at rt. Then cellulose sheets were washed with THF (2 × 30 mL), acetone (2 × 30 mL), acetone:H_2_O 1:1 (2 × 40 mL), acetone (2 × 40 mL) and with DCM (30 mL), than left to remove the remaining solvent and dried in a vacuum desiccator. Elemental analysis: for cellulose: %N 0.00–0.05, %Cl 0.01–0.05, after DCMT immobilization found: %N 3.64, %Cl 2.66. The loading of the cellulose sheets with triazine was calculated from elemental analysis data. The surface loading was calculated according to nitrogen content 31.9 × 10^−6^ mol (N)/cm^2^, which was equivalent to NL^s^ = 10.6×10^−6^ mol (triazine)/cm^2^ and ClL^w^ = 0.75 mmol (Cl)/1 g, giving equivalent to ClL^s^ = 9.2×10^−6^ mol (Cl)/cm^2^. Immobilization of *m*-phenylenediamine: The cellulose sheets functionalized with DCMT were immersed in 1 M solution of *m*-phenylenediamine (4.3 g) in THF (40 mL) and gently shaken for 24 h at rt. Then sheets were washed successively with THF (2 × 20 mL), DMF (2 × 20 mL) and THF (2 × 20 mL) again and dried in the vacuum desiccator. SPOT synthesis of *N*-lipidated peptides on the cellulose: The peptides were synthesized on an automatic synthesizer ResPep SL by Intavis using a modified SPOT procedure [45]. The 0.56 M solutions of all Fmoc-protected natural amino acids were prepared in (*N*-methyl-2-pyrrolidone) (NMP). As a coupling reagent, a 0.5 M solution of 4-(4,6-dimethoxy-1,3,5-triazin-2-yl)-4-methylmorpholinium 4-toluenesulfonate (DMT/NMM/TosO^−^) in DMF was used. As a base, 2 M NMM in DMF was used. To remove Fmoc protecting group, 25% piperidine in DMF was used. The first synthetic cycle included: Preactivation: 6 min, for each spot: 0.15 μL solution of coupling reagent; 0.073 μL solution of NMM; 0.17 μL amino acid (Fmoc-Ala-OH, Fmoc-Pro-OH, Fmoc-Phe-OH); 0.003 μL NMP; Coupling: 2 × 3 min; for each coupling, 0.198 μL of the preactivated solution was used. The second synthetic cycle included: Fmoc-group deprotection (2 × 10 min, dispense volume: 500 μL); Preactivation: 6 min, for each spot: 0.15 μL solution of coupling reagent; 0.073 μL solution of NMM; 0.17 μL amino acid (all natural amino acids); 0.003 μL NMP; Coupling: 2 × 3 min; for each coupling, 0.198 μL of preactivated solution is used. The third synthetic cycle included: Fmoc-group deprotection (2 × 10 min, dispense volume: 500 μL); Coupling: 1 × 7.5 min; for each spot were used: 0.3 μL NMP and 0.15 μL of 0.25 M solution of triazine esters of heptanoic acid and decanoic acid in DMF:NMP (1:1). Deprotection of the side chains of dipeptides: Modified cellulose membranes were treated with a mixture consisting of 50% (*v*/*v*) trifluoroacetic acid in DCM (200 mL) with 3% (*v*/*v*) water and 2% (*v*/*v*) trisopropylsilane for 5 h. Then the membranes were washed with DCM (2 × 100 mL), EtOH (2 × 100 mL) and dried in a vacuum desiccator. Buffering of cellulose membranes: The modified cellulose membrane was buffered with phosphate buffer pH 7.0 (200 mL) for 30 min. Then, celluloses were washed with water (2 × 200 mL) and a mixture MeOH:H_2_O (1:1) and dried to constant weight in the vacuum dessicator. During the synthesis, two identical libraries were prepared on each functionalized sheet, after splitting each sheet for two parts, one of them was treated with the active substance and then with the reporter dye (Brilliant Black), the second one was treated with the reporter dye only.

### 3.6. Anticancer Compounds Docking

The dot arrays consisting of a library of artificial peptide receptors were buffered (phosphate buffer, pH = 7) and conditioned in a vacuum desiccator overnight before being immersed into a gently shaken solution (25 mL) of anticancer active ligands in MeOH (25 μmol/100 mL) for 15 min. The library was then washed four times with MeOH (50 mL) and dried in the vacuum desiccator. After conditioning, the library was treated with a 25 mL solution of the reporter dye (Brilliant Black, 100 μmol/100 mL MeOH) for 15 min. Any excess of the reporter dye was removed by successive washings with methanol (5 × 50 mL), which were continued until a colourless washing solvent was obtained, after which they were dried in the vacuum desiccator. All of the structures were scanned in the 24-bit RGB colour space. The resulting scans were converted into 256-bit grey scale and processed using the Image Quant program. The average value of grey coloration was determined for all of the discs in library in this way. Ability of molecular receptors to interact with colorless active substances (*I_D_*) was calculated as difference in intensity of coloration by reporter dye (*I_Drd_*) and intensity of coloration with active substance and then with reporter dye (Brilliant Black) (*I_Da_*).

*I_D_* = *I_Drd_* − *I_Da_* (7)

where: *I_D_*—intensity of ligand binding measured by coloration in 256 bit gray scale, *I_Drd_*—intensity of coloration with reporter dye, *I_Da_*—intensity of dyeing after treatment of disk with active substance and subsequently with reporter dye. Thus, the *I_D_* is proportional to the amount of ligand bound to the receptor.

### 3.7. Binding of Anticancer Active Ligands to Molecular Receptors Pockets

The result of docking of compounds with antitumor activity into the molecular receptor binding cavities is shown in the form of an intensity binding map (Figure 4b). The research began with the search for molecular receptors that form strong complexes (no displacement from the binding cavity under the action of a reporter compound) with all docked derivatives. This assumption was due to the fact that the common element of all tested compounds (with exception of compounds **15** and **20**) is the presence of a methoxy substituent and the *N*-(2-chloroethyl)piperazine moiety on the triazine ring. Finding molecular receptors binding all compounds can provide information about the amino acid structure of the peptide receptor fragment responsible for the formation of molecular targets. For a sublibrary containing alanine as a C-terminal amino acid, receptors binding all docked compounds were RA, KA, SA, TA, YA, IA, LA, VA and PA. In all cases, the selected receptors were lipidated at the *N*-terminal position with *n*-decanoic acid residue. For a sublibrary containing phenylalanine at the C-terminal position, receptors able to interact with all docked ligands were *n*-heptanoyl-AF, *n*-decanoyl-PF, *n*-decanoyl-KF, *n*-decanoyl-TF, *n*-decanoyl-IF, *n*-decanoyl-YF, *n*-decanoyl-LF and *n*-decanoyl-VF. In the case of a sublibrary containing proline in the C-terminal position, the following receptors were selected: NP, TP, GP, VP, IP. In all cases, the generated receptors were lipidated at the *N*-terminal position with an *n*-decanoic acid (D) residue.

### 3.8. Model Builder & Molecular Modelling

**Figure 9:**
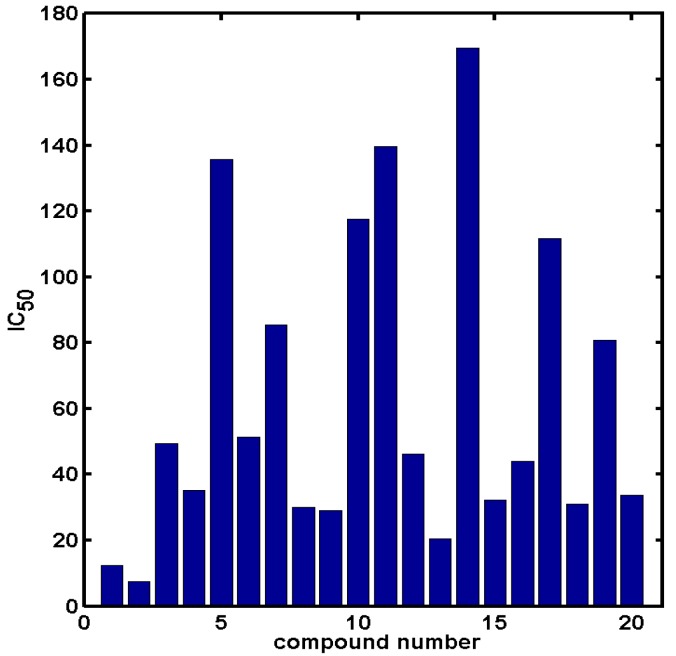
In vitro IC_50_ activity for set of anticancer analogues.

The same laboratory was employed to specify inhibitory activity data (IC_50_) to eliminate potential data noise that might be introduced by pooling of data sets coming from various sources. The structures and the in vitro efficiency of derivatives containing the *N*-chloroethyl-piperazine scaffold attached directly to the 1,3,5-triazine moiety are introduced in Table 1 and Figure 9, respectively. The CACTVS/csed molecular editor was used to specify the constitution of the respective compound models [46]. The spatial geometry of molecules was produced using the 3D generator CORINA [47]. The (inter)change file format converter OpenBabel was applied to convert the chemical data [48]. The principal components of the modelling studies were conducted with the usage of the Sybyl-X 2.0/Certara software package running on a HP workstation with Debian 6.0 operating system [49].

The standard Tripos force field (POWELL conjugate gradient algorithm) with 0.01 kcal/mol energy gradient convergence criterion and a distant dependent dielectric constant was used to optimize the initial geometry of each compound (MAXMIN2 module). The Gasteiger-Hückel procedure implemented in Sybyl for the electrostatic potential calculations was initially employed to calculate the partial atomic charges. The trial alignments are typically defined to systematically span the common scaffold of the analysed compounds; therefore, one 15-ordered atom trial alignment on molecule **2** according to the active analogue approach (AAA) was selected to cover the entire bonding topology in the maximal common structure (conjugated triazine/piperazine/chloroethyl motif) by atom FIT method based on the matching of atom positions between the corresponding atom pairs (1–15). The sp^3^ hybridized carbon probe atom with the charge of +1 and 0 and hydrogen as a probe atom with the charge of +1 were used to calculate the electrostatic and steric fields. The CoMFA grid spacing was 2.0 Å for all Cartesian dimensions within the defined region of the 3D lattice, which extended beyond the van der Waals envelopes of all molecules by at least 4.0 Å. Non-covalent interaction fields were specified at each intersection on the regularly spaced grid of points. For each molecule, energies with the total of 1820 grid points were calculated with 2 Å spacing in a 14 × 10 × 13 lattice. All columns with energy variance less than 2.0 kcal/mol were discarded by setting the sigma parameter to 2.0 kcal/mol to reduce data noise. Both steric/electrostatic energies with a value greater than 30.0 kcal/mol were truncated to a tentative value of 30.0 (default cutoff). The SAMPLS method with standard internal and external validation techniques was used to determine the statistical index of the model predictive power describing the variations in CoMFA interaction fields (independent variables) and changes in activity (dependent variable). The SONNIA software was employed in the CoMSA analysis to simulate 20 × 20 to 50 × 50 SOMs with the winning distance (md) varyiing in the range of 0.2–2.0. The Cartesian coordinates of molecular surfaces for superimposed molecules were processed by the SOM network to form a two-dimensional map of electrostatic potential—the most active analogue **2** was used to form a template molecule [50]. The output maps were subsequently transformed to a 400- or 2500-element vector which was processed by the PLS method implemented in the MATLAB programming environment in correspondence with the CoMFA analysis [51].

## 4. Conclusions

The general pattern of the anticancer compounds comprises the *N*-(2-chloroethyl)piperazine moiety and a methoxy substituent on the triazine ring. In our case, almost all investigated compounds contained two amino substituents mimicking the structure of 2,4,6-amino-1,3,5-triazine derivatives with various amino substituents; therefore, the following chemotypes can be distinquised: derivatives containing aromatic amine residues, aliphatic amines, amino acid residues, dipeptide derivatives and derivatives containing two moieties *N*-(2-chloroethyl)piperazine and aromatic amine residues or the methoxy substituent. Moreover, the examined molecules can also be subdivided in relation to the hydrophobicity and hydrophilicity. The pharmacophore properties of a new series of potential P2X inhibitors using a coupled neural network and the IVE-PLS method were examined in the ligand-based comparative molecular surface analysis. The observed inhibitory potency was correlated with surface descriptors regarding multiple training/test populations. In particular, some regions with suggested unfavourable contribution appear in the close proximity to the negatively charged oxygen atom and hydrogens of the metoxy group directly attached to the triazine motif. The importance of the area occupied by the positively charged hydrogen atom bonded to the nitrogen atom was demonstrated as a significant factor interacting non-covalently with the macromolecular site (hydrogen bond donor). Moreover, the PCA procedure was employed for the set of Dragon and Sybyl descriptors to analyse the dataset (dis)similarity. A focus was made on the interpretation of noticeable variations in the potential selectiveness of individual inhibitor-receptor interactions due to their physicochemical properties; therefore, the library of artificial receptors was designed and examined. The resulting library response to individual inhibitors was arranged in the array, preprocessed and transformed by the PCA and PLS procedures. A dominant absolute contribution of Glu attached to heptanoic gating acid and Phe bonded to the linker *m*-phenylenediamine/triazine scaffold to PC1 was revealed by the PCA analysis. Comparable results were obtained while correlating the ADR response with experimental activity data. The IVE-PLS procedure indicated receptor systems with predominant Pro bonded to the linker and Glu, Gln, Cys and Val directly attached to the gating acid. The direct specification of Glu/Gln residues in ligand binding suggests the potential involvement in the hydrogen bonding, for instance, with hydrogen attached to the nitrogen atom as indicated in the CoMSA study. Val contributes to the hydrophobic environment of the binding pocket, while Phe can interact with the ligand aromatic ring via π-stacking phenomena. Interestingly, substituting Gln with Cys increases the P2X receptor sensitivity due to the potential generation of disulfide bridges important for the gating mechanism. The proposed comprehensive ligand-based and simplified structure-based methodology allows the in-depth study of the performance of peptide receptors against the tested set of compounds. In particular, an insight into the selectivity of the library can be gained, while increasing the number of amino residues attached (polypeptide population), and therefore, it will be easier to indicate possible new directions towards intelligent drug delivery systems.

## Figures and Tables

**Table 1:** Structure and activity of host molecules [13,16,17]. IC_50_ values were determined experimentally on the MCF-7 cell line.

Comp.	Compound Structure	IC_50_ [μM]	Comp.	Compound Structure	IC_50_ [μM]
**1**	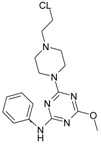	12.30	**11**	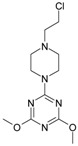	139.78
**2**	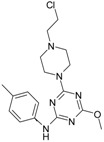	7.40	**12**	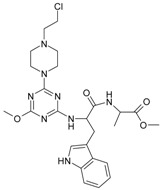	46.29
**3**	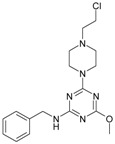	49.40	**13**	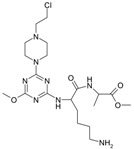	20.44
**4**	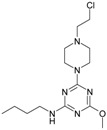	35.11	**14**	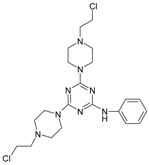	169.7
**5**	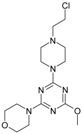	135.73	**15**	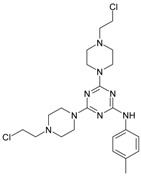	32.14
**6**	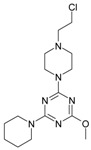	51.39	**16**	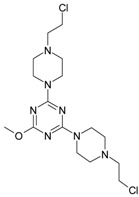	43.95
**7**	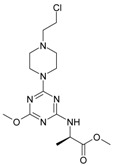	85.37	**17**	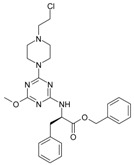	111.81
**8**	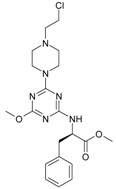	30.02	**18**	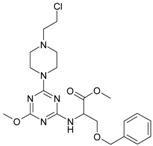	30.92
**9**	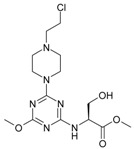	29.08	**19**	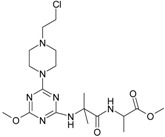	80.83
**10**	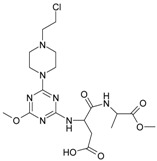	117.61	**20**	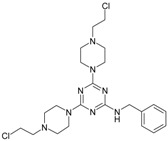	33.85

## Supplementary Materials

The supplementary material is available online. Table S1: Molecular descriptors calculated with Sybyl software, Table S2: Position on cellulose sheet and structure of array of artificial peptide receptors, Figure S1: Projection of variables on plane defined by first and second loadings for Dragon (a) and Sybyl (b) parameters.
