# Supplementary material for: Towards Intelligent Drug Design System: Application of Artificial Dipeptide Receptor Library in QSAR-Oriented Studies
Source: Molecules. 2018 Aug 6;23(8):1964. doi: 10.3390/molecules23081964 (PMC6222794; doi:10.3390/molecules23081964)
Supplement: Supplementary file 1 [file molecules-23-01964-s001.pdf]

## Supplementary Materials

# Towards Intelligent Drug Design System: Application of Artificial Dipeptide Receptor Library in QSAR-Oriented Studies

**Andrzej Bak [1,*], Violetta Kozik [1], Malgorzata Walczak [2], Justyna Fraczyk [2], Zbigniew Kaminski [2], Beata Kolesinska [2], Adam Smolinski [3], Josef Jampilek [4,*]**

[1] Department of Synthesis Chemistry, Faculty of Mathematics, Physics and Chemistry, University of Silesia, Szkolna 9, 40007 Katowice, Poland; violetta.kozik@us.edu.pl

[2] Institute of Organic Chemistry, Lodz University of Technology, Zeromskiego 116, 90924 Lodz, Poland; malgorzata.walczak@p.lodz.pl (M.W.), justyna.fraczyk@p.lodz.pl (J.F.), zbigniew.kaminski@p.lodz.pl (Z.K.), beata.kolesinska@p.lodz.pl (B.K.)

[3] Central Mining Institute, Gwarkow 1, 40166 Katowice, Poland; asmolinski@gig.katowice.pl (A.S.)

[4] Department of Pharmaceutical Chemistry, Faculty of Pharmacy, Comenius University, Odbojarov 10, 83232 Bratislava, Slovakia

* Correspondence: andrzej.bak@us.edu.pl (A.B.); josef.jampilek@gmail.com (J.J.)

**Table S1.** Molecular descriptors calculated with Sybyl software.

| L.p. | polar surface area | volume | polar volume | acceptor count | clogP | donor count | Ro5 violations | molecular weight | rotatable bond count |
|---|---|---|---|---|---|---|---|---|---|
| **1.** | 63.824 | 1058.514 | 297.659 | 7 | 3.9438 | 1 | 0 | 348.8305 | 6 |
| **2.** | 63.913 | 1113.909 | 294.349 | 7 | 4.4428 | 1 | 0 | 362.8571 | 6 |
| **3.** | 59.635 | 1121.490 | 307.347 | 7 | 3.4128 | 1 | 0 | 362.8571 | 7 |
| **4.** | 59.223 | 1072.394 | 313.306 | 7 | 3.5518 | 1 | 0 | 328.8409 | 8 |
| **5.** | 49.670 | 1030.942 | 300.050 | 8 | 1.2955 | 0 | 0 | 342.8244 | 5 |
| **6.** | 34.303 | 1055.849 | 292.437 | 7 | 2.6775 | 0 | 0 | 340.8516 | 5 |
| **7.** | 83.034 | 1083.036 | 339.623 | 9 | 2.0264 | 1 | 0 | 358.8238 | 8 |
| **8.** | 71.756 | 1297.145 | 323.532 | 9 | 3.4444 | 1 | 0 | 434.9198 | 10 |
| **9.** | 127.346 | 1124.004 | 374.276 | 10 | 0.7882 | 2 | 0 | 374.8232 | 9 |
| **10.** | 183.253 | 1331.860 | 425.484 | 13 | -1.1538 | 3 | 0 | 473.9112 | 12 |
| **11.** | 56.549 | 867.528 | 275.754 | 7 | 2.0154 | 0 | 0 | 287.7459 | 5 |
| **12.** | 118.914 | 1479.006 | 440.206 | 12 | 3.1768 | 3 | 1 | 545.0337 | 12 |
| **13.** | 176.800 | 1470.734 | 409.156 | 12 | 1.4688 | 3 | 0 | 486.9961 | 14 |
| **14.** | 42.977 | 1338.142 | 315.506 | 8 | 4.3696 | 1 | 0 | 465.4225 | 8 |
| **15.** | 43.030 | 1390.771 | 302.718 | 8 | 4.7496 | 1 | 0 | 479.4491 | 8 |
| **16.** | 34.469 | 1201.483 | 341.483 | 8 | 2.3295 | 0 | 0 | 404.3379 | 7 |
| **17.** | 70.710 | 1452.356 | 343.600 | 9 | 5.1552 | 1 | 2 | 511.0157 | 12 |
| **18.** | 74.499 | 1342.959 | 335.740 | 10 | 3.2898 | 1 | 0 | 464.9458 | 12 |
| **19.** | 95.669 | 1306.901 | 408.794 | 11 | 2.0778 | 2 | 0 | 443.9283 | 10 |
| **20.** | 38.187 | 1444.600 | 337.463 | 8 | 3.8386 | 1 | 0 | 479.4491 | 9 |

**Table S2.** Position on cellulose sheet and structure of array of artificial peptide receptors.

| Position | ADP structure |
|---|---|
| **A1** | n-decanoyl-AA-NH-$C_6H_4$-NH-DMT-cellulose |
| **A2** | n-heptanoyl-AA-NH-$C_6H_4$-NH-DMT-cellulose |
| **A3** | n-decanoyl-RA-NH-$C_6H_4$-NH-DMT-cellulose |
| **A4** | n-heptanoyl-RA-NH-$C_6H_4$-NH-DMT-cellulose |
| **A5** | n-decanoyl-NA-NH-$C_6H_4$-NH-DMT-cellulose |
| **A6** | n-heptanoyl-NA-NH-$C_6H_4$-NH-DMT-cellulose |
| **A7** | n-decanoyl-DA-NH-$C_6H_4$-NH-DMT-cellulose |
| **A8** | n-heptanoyl-DA-NH-$C_6H_4$-NH-DMT-cellulose |
| **A9** | n-decanoyl-CA-NH-$C_6H_4$-NH-DMT-cellulose |
| **A10** | n-heptanoyl-CA-NH-$C_6H_4$-NH-DMT-cellulose |
| **A11** | n-decanoyl-QA-NH-$C_6H_4$-NH-DMT-cellulose |
| **A12** | n-heptanoyl-QA-NH-$C_6H_4$-NH-DMT-cellulose |
| **A13** | n-decanoyl-EA-NH-$C_6H_4$-NH-DMT-cellulose |
| **A14** | n-heptanoyl-EA-NH-$C_6H_4$-NH-DMT-cellulose |
| **A15** | n-decanoyl-GA-NH-$C_6H_4$-NH-DMT-cellulose |
| **A16** | n-heptanoyl-GA-NH-$C_6H_4$-NH-DMT-cellulose |
| **A17** | n-decanoyl-HA-NH-$C_6H_4$-NH-DMT-cellulose |
| **A18** | n-heptanoyl-HA-NH-$C_6H_4$-NH-DMT-cellulose |
| **A19** | n-decanoyl-IA-NH-$C_6H_4$-NH-DMT-cellulose |
| **A20** | n-heptanoyl-IA-NH-$C_6H_4$-NH-DMT-cellulose |
| **B1** | n-decanoyl-LA-NH-$C_6H_4$-NH-DMT-cellulose |
| **B2** | n-heptanoyl-LA-NH-$C_6H_4$-NH-DMT-cellulose |
| **B3** | n-decanoyl-KA-NH-$C_6H_4$-NH-DMT-cellulose |
| **B4** | n-heptanoyl-KA-NH-$C_6H_4$-NH-DMT-cellulose |
| **B5** | n-decanoyl-MA-NH-$C_6H_4$-NH-DMT-cellulose |
| **B6** | n-heptanoyl-MA-NH-$C_6H_4$-NH-DMT-cellulose |
| **B7** | n-decanoyl-FA-NH-$C_6H_4$-NH-DMT-cellulose |
| **B8** | n-heptanoyl-FA-NH-$C_6H_4$-NH-DMT-cellulose |
| **B9** | n-decanoyl-PA-NH-$C_6H_4$-NH-DMT-cellulose |
| **B10** | n-heptanoyl-PA-NH-$C_6H_4$-NH-DMT-cellulose |
| **B11** | n-decanoyl-SA-NH-$C_6H_4$-NH-DMT-cellulose |
| **B12** | n-heptanoyl-SA-NH-$C_6H_4$-NH-DMT-cellulose |
| **B13** | n-decanoyl-TA-NH-$C_6H_4$-NH-DMT-cellulose |
| **B14** | n-heptanoyl-TA-NH-$C_6H_4$-NH-DMT-cellulose |
| **B15** | n-decanoyl-WA-NH-$C_6H_4$-NH-DMT-cellulose |
| **B16** | n-heptanoyl-WA-NH-$C_6H_4$-NH-DMT-cellulose |
| **B17** | n-decanoyl-YA-NH-$C_6H_4$-NH-DMT-cellulose |
| **B18** | n-heptanoyl-YA-NH-$C_6H_4$-NH-DMT-cellulose |
| **B19** | n-decanoyl-VA-NH-$C_6H_4$-NH-DMT-cellulose |
| **B20** | n-heptanoyl-VA-NH-$C_6H_4$-NH-DMT-cellulose |
| **C1** | n-decanoyl-AF-NH-$C_6H_4$-NH-DMT-cellulose |
| **C2** | n-heptanoyl-AF-NH-$C_6H_4$-NH-DMT-cellulose |
| **C3** | n-decanoyl-RF-NH-$C_6H_4$-NH-DMT-cellulose |
| **C4** | n-heptanoyl-RF-NH-$C_6H_4$-NH-DMT-cellulose |
| **C5** | n-decanoyl-NF-NH-$C_6H_4$-NH-DMT-cellulose |
| **C6** | n-heptanoyl-NF-NH-$C_6H_4$-NH-DMT-cellulose |
| **C7** | n-decanoyl-DF-NH-$C_6H_4$-NH-DMT-cellulose |
| **C8** | n-heptanoyl-DF-NH-$C_6H_4$-NH-DMT-cellulose |
| **C9** | n-decanoyl-CF-NH-$C_6H_4$-NH-DMT-cellulose |
| **C10** | n-heptanoyl-CF-NH-$C_6H_4$-NH-DMT-cellulose |
| **C11** | n-decanoyl-QF-NH-$C_6H_4$-NH-DMT-cellulose |

| | |
|---|---|
| **C12** | n-heptanoyl-QF-NH-$C_6H_4$-NH-DMT-cellulose |
| **C13** | n-decanoyl-EF-NH-$C_6H_4$-NH-DMT-cellulose |
| **C14** | n-heptanoyl-EF-NH-$C_6H_4$-NH-DMT-cellulose |
| **C15** | n-decanoyl-GF-NH-$C_6H_4$-NH-DMT-cellulose |
| **C16** | n-heptanoyl-GF-NH-$C_6H_4$-NH-DMT-cellulose |
| **C17** | n-decanoyl-HF-NH-$C_6H_4$-NH-DMT-cellulose |
| **C18** | n-heptanoyl-HF-NH-$C_6H_4$-NH-DMT-cellulose |
| **C19** | n-decanoyl-IF-NH-$C_6H_4$-NH-DMT-cellulose |
| **C20** | n-heptanoyl-IF-NH-$C_6H_4$-NH-DMT-cellulose |
| **D1** | n-decanoyl-LF-NH-$C_6H_4$-NH-DMT-cellulose |
| **D2** | n-heptanoyl-LF-NH-$C_6H_4$-NH-DMT-cellulose |
| **D3** | n-decanoyl-KF-NH-$C_6H_4$-NH-DMT-cellulose |
| **D4** | n-heptanoyl-KF-NH-$C_6H_4$-NH-DMT-cellulose |
| **D5** | n-decanoyl-MF-NH-$C_6H_4$-NH-DMT-cellulose |
| **D6** | n-heptanoyl-MF-NH-$C_6H_4$-NH-DMT-cellulose |
| **D7** | n-decanoyl-FF-NH-$C_6H_4$-NH-DMT-cellulose |
| **D8** | n-heptanoyl-FF-NH-$C_6H_4$-NH-DMT-cellulose |
| **D9** | n-decanoyl-PF-NH-$C_6H_4$-NH-DMT-cellulose |
| **D10** | n-heptanoyl-PF-NH-$C_6H_4$-NH-DMT-cellulose |
| **D11** | n-decanoyl-SF-NH-$C_6H_4$-NH-DMT-cellulose |
| **D12** | n-heptanoyl-SF-NH-$C_6H_4$-NH-DMT-cellulose |
| **D13** | n-decanoyl-TF-NH-$C_6H_4$-NH-DMT-cellulose |
| **D14** | n-heptanoyl-TF-NH-$C_6H_4$-NH-DMT-cellulose |
| **D15** | n-decanoyl-WF-NH-$C_6H_4$-NH-DMT-cellulose |
| **D16** | n-heptanoyl-WF-NH-$C_6H_4$-NH-DMT-cellulose |
| **D17** | n-decanoyl-YF-NH-$C_6H_4$-NH-DMT-cellulose |
| **D18** | n-heptanoyl-YF-NH-$C_6H_4$-NH-DMT-cellulose |
| **D19** | n-decanoyl-VF-NH-$C_6H_4$-NH-DMT-cellulose |
| **D20** | n-heptanoyl-VF-NH-$C_6H_4$-NH-DMT-cellulose |
| **E1** | n-decanoyl-AP-NH-$C_6H_4$-NH-DMT-cellulose |
| **E2** | n-heptanoyl-AP-NH-$C_6H_4$-NH-DMT-cellulose |
| **E3** | n-decanoyl-RP-NH-$C_6H_4$-NH-DMT-cellulose |
| **E4** | n-heptanoyl-RP-NH-$C_6H_4$-NH-DMT-cellulose |
| **E5** | n-decanoyl-NP-NH-$C_6H_4$-NH-DMT-cellulose |
| **E6** | n-heptanoyl-NP-NH-$C_6H_4$-NH-DMT-cellulose |
| **E7** | n-decanoyl-DP-NH-$C_6H_4$-NH-DMT-cellulose |
| **E8** | n-heptanoyl-DP-NH-$C_6H_4$-NH-DMT-cellulose |
| **E9** | n-decanoyl-CP-NH-$C_6H_4$-NH-DMT-cellulose |
| **E10** | n-heptanoyl-CP-NH-$C_6H_4$-NH-DMT-cellulose |
| **E11** | n-decanoyl-QP-NH-$C_6H_4$-NH-DMT-cellulose |
| **E12** | n-heptanoyl-QP-NH-$C_6H_4$-NH-DMT-cellulose |
| **E13** | n-decanoyl-EP-NH-$C_6H_4$-NH-DMT-cellulose |
| **E14** | n-heptanoyl-EP-NH-$C_6H_4$-NH-DMT-cellulose |
| **E15** | n-decanoyl-GP-NH-$C_6H_4$-NH-DMT-cellulose |
| **E16** | n-heptanoyl-GP-NH-$C_6H_4$-NH-DMT-cellulose |
| **E17** | n-decanoyl-HP-NH-$C_6H_4$-NH-DMT-cellulose |
| **E18** | n-heptanoyl-HP-NH-$C_6H_4$-NH-DMT-cellulose |
| **E19** | n-decanoyl-IP-NH-$C_6H_4$-NH-DMT-cellulose |
| **E20** | n-heptanoyl-IP-NH-$C_6H_4$-NH-DMT-cellulose |
| **F1** | n-decanoyl-LP-NH-$C_6H_4$-NH-DMT-cellulose |
| **F2** | n-heptanoyl-LP-NH-$C_6H_4$-NH-DMT-cellulose |
| **F3** | n-decanoyl-KP-NH-$C_6H_4$-NH-DMT-cellulose |
| **F4** | n-heptanoyl-KP-NH-$C_6H_4$-NH-DMT-cellulose |
| **F5** | n-decanoyl-MP-NH-$C_6H_4$-NH-DMT-cellulose |
| **F6** | n-heptanoyl-MP-NH-$C_6H_4$-NH-DMT-cellulose |

| | |
|---|---|
| **F7** | n-decanoyl-FP-NH-$C_6H_4$-NH-DMT-cellulose |
| **F8** | n-heptanoyl-FP-NH-$C_6H_4$-NH-DMT-cellulose |
| **F9** | n-decanoyl-PP-NH-$C_6H_4$-NH-DMT-cellulose |
| **F10** | n-heptanoyl-PP-NH-$C_6H_4$-NH-DMT-cellulose |
| **F11** | n-decanoyl-SP-NH-$C_6H_4$-NH-DMT-cellulose |
| **F12** | n-heptanoyl-SP-NH-$C_6H_4$-NH-DMT-cellulose |
| **F13** | n-decanoyl-TP-NH-$C_6H_4$-NH-DMT-cellulose |
| **F14** | n-heptanoyl-TP-NH-$C_6H_4$-NH-DMT-cellulose |
| **F15** | n-decanoyl-WP-NH-$C_6H_4$-NH-DMT-cellulose |
| **F16** | n-heptanoyl-WP-NH-$C_6H_4$-NH-DMT-cellulose |
| **F17** | n-decanoyl-YP-NH-$C_6H_4$-NH-DMT-cellulose |
| **F18** | n-heptanoyl-YP-NH-$C_6H_4$-NH-DMT-cellulose |
| **F19** | n-decanoyl-VP-NH-$C_6H_4$-NH-DMT-cellulose |
| **F20** | n-heptanoyl-VP-NH-$C_6H_4$-NH-DMT-cellulose |

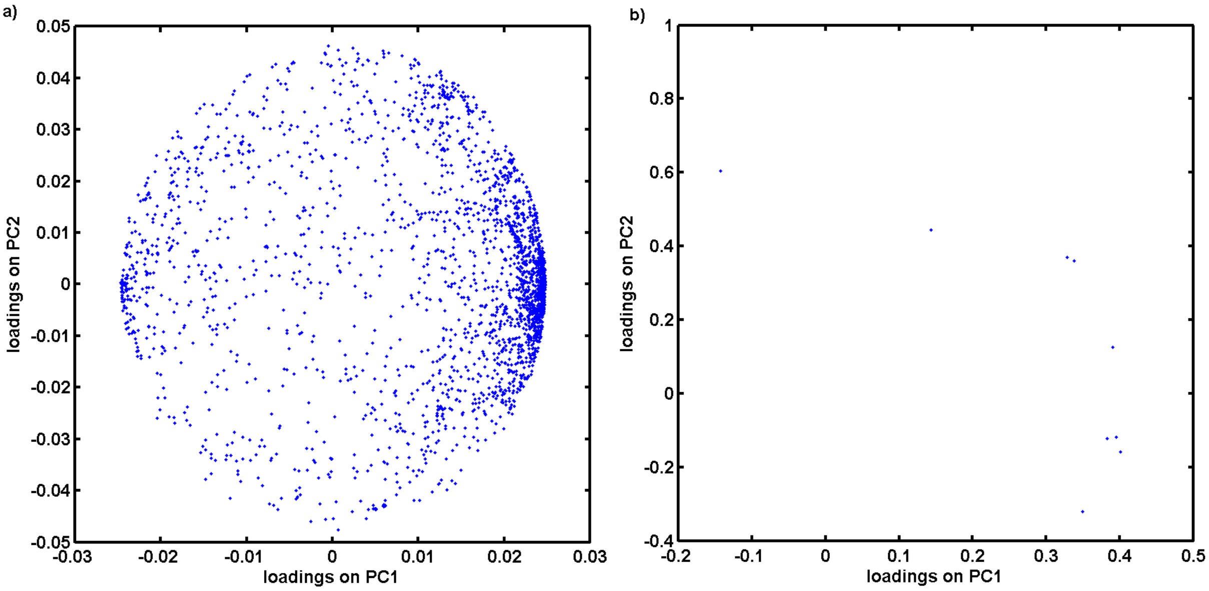


**Figure S1.** Projection of variables on plane defined by first and second loadings for Dragon (a) and Sybyl (b) parameters.
